# Supplementary material for: Multiparametric cellular and spatial organization in cancer tissue lesions with a streamlined pipeline
Source: Nat Biomed Eng. 2025 Aug 25;10(3):517–31. doi: 10.1038/s41551-025-01475-9 (PMC13008776; doi:10.1038/s41551-025-01475-9)
Supplement: Supplementary file 1 — Supplementary Tables 1–3 and Figs. 1–8. [file 41551_2025_1475_MOESM1_ESM.pdf]

nature biomedical engineering

Supplementary information

https://doi.org/10.1038/s41551-025-01475-9

Multiparametric cellular and spatial organization in cancer tissue lesions with a streamlined pipeline

In the format provided by the authors and unedited

Integrative filetypes with third-party applications		Technologies that MARQO can analyze
Third-party files that can be imported into MARQO		*Tested and validated*
.ome.tiff	.jpeg	Multiplex immunohistochemical consecutive staining on a single slide (MICSSS)[5]
.tiff	.png	COMET multiplex immunofluorescence (mIF)[9]
.svs	.vms	Singleplex IHC
.ndpi	.nd2	
MARQO outputs that can be exported to third-party applications		*Tested*
.geojson		Orion[10]
.ome.tiff		CODEX[1]
.csv		Cyclic immunofluorescence (CyCIF)[2]

Supplementary Table 1: MARQO compatibility to import and export files with third-party applications (left) and its validation on multiple staining technologies (right).

Cycle	Marker	Detection chromogen	Antibody clone	Antibody supplier	Catalog #	Antibody Dilution	Antibody incubation time (min)	Chromogen incubation time (min)
Singleplex IHC								
1	CD3	DAB	LN10	Leica Biosystems	PA0553-U	RTU	15	10
1	PD-L1	AEC	E1L3N	Cell Signaling Technology	13684S	1/400	15	15
MICSSS on TMA								
1	PD-L1	AEC	E1L3N	Cell Signaling Technology	13684S	1/400	15	15
2	PD-1	AEC	EPR4877(2)	Abcam	ab137132	1/250	15	15
3	CD8	AEC	C8/144b	Dako	M7103	1/100	15	3
4	CD3	AEC	2GV6	Ventana	790-4341	RTU	15	3
5	PanCK	AEC	AE1/AE3	Dako	M351501-2	1/50	15	15
6	FoxP3	AEC	236A/E7	Abcam	ab20034	1/80	15	15
MICSSS on biopsy and whole-slide HCC resections								
1	PD-1	AEC	EPR4877(2)	Abcam	ab137132	1/250	15	15
2	FoxP3	AEC	236A/E7	Abcam	ab20034	1/80	15	15
3	CD3	AEC	2GV6	Ventana	PA0553-U	RTU	15	15
4	CD8	AEC	C8/144b	Dako	M7103	1/100	15	15
5	Ki-67	AEC	30-9	Ventana	790-4286	RTU	15	15
6	αSMA	AEC	1A4	Dako	M085101-2	1/250	15	15
7	CD68	AEC	KP1	Dako	M081401-2	1/1000	15	15
8	CD20	AEC	L26	Dako	M075501-2	1/250	15	15
9	PanCK	AEC	AE1/AE3	Dako	M351501-2	1/50	15	15
10	MZB1	AEC	Polyclonal	Sigma Aldrich	HPA043745	1/5000	15	15
MICSSS on whole-slide NSCLC resections								
1	PD-L1	AEC	E1L3N	Cell Signaling Technology	ab137132	1/400	15	15
2	FoxP3	AEC	236A/E7	Abcam	ab20034	1/80	15	15
3	PD1	AEC	EPR4877(2)	Abcam	ab137132	1/250	15	15
4	aSMA	AEC	1A4	Dako	M085101-2	1/250	15	5
5	CD68	AEC	KP1	Dako	M081401-2	1/1000	15	5
6	Ki-67	AEC	30-9	Ventana	790-4286	RTU	15	3
7	CD8	AEC	C8/144b	Dako	M7103	1/100	15	3
8	CD3	AEC	2GV6	Ventana	790-4341	RTU	15	3
9	CD20	AEC	L26	Dako	M075501-2	1/250	15	5
10	PanCK	AEC	AE1/AE3	Dako	M351501-2	1/50	15	15

Supplementary Table 2: Primary antibodies used for singleplex IHC, MICSSS on TMA, and MICSSS on biopsy and whole-slide HCC and NSCLC resections. **RTU = Ready-to-use*

Cycle	Marker	Detection channel	Primary Antibodies						Secondary Antibodies					
			Clone	Supplier	Catalog #	Lot #	Dilution	Incubation time (min)	Antibody	Supplier	Catalog #	Lot #	Dilution	Incubation time (min)
1	**CD11c**	TRITC	EP1347Y	abcam	ab52632	GR3334379-2	1:2400	8	Alexa Fluor™ Plus 555 goat anti-rabbit	Thermo Fisher Scientific	A32732	WL332194	1:100	2
	**LAG-3**	Cy5	17B4	Novus Biologicals	NBP1-97657	8052108	1:50		Alexa Fluor™ Plus 647 goat anti-mouse	Thermo Fisher Scientific	A32733	WC310044	1:200	2
2	**CK**	TRITC	AE1/AE3	Dako	M351501-2	11313302	1:150	4	Alexa Fluor™ Plus 555 goat anti-mouse	Thermo Fisher Scientific	A32727	WL333735	1:100	2
	**FOXP3**	Cy5	BLR034F	Bethyl	A700-034	#2	1:75		Alexa Fluor™ Plus 647 goat anti-rabbit	Thermo Fisher Scientific	A32733	WC310044	1:200	2
3	**CD3**	TRITC	MRQ-39	CellMarque	103R-96	0000113203	1:300	4	Alexa Fluor™ Plus 555 goat anti-rabbit	Thermo Fisher Scientific	A32732	WL332194	1:100	2
	**CD8**	Cy5	4B11	BioRad	MCA1817	158759	1:125		Alexa Fluor™ Plus 647 goat anti-mouse	Thermo Fisher Scientific	A32728	WK331591	1:200	2
4	**CD11b**	TRITC	EPR1344	abcam	ab133357	GR3276764-5	1:1500	4	Alexa Fluor™ Plus 555 goat anti-rabbit	Thermo Fisher Scientific	A32732	WL332194	1:100	2
	**Ki67**	Cy5	MIB-1	Dako	M724029-2	41327889	1:100		Alexa Fluor™ Plus 647 goat anti-mouse	Thermo Fisher Scientific	A32728	WK331591	1:200	2
5	**CD68**	TRITC	KP1	Thermo	MA5-13324	2347179	1:250	4	Alexa Fluor™ Plus 555 goat anti-mouse	Thermo Fisher Scientific	A32727	WL333735	1:100	2
	**CD4**	Cy5	EPR6855	abcam	ab133616	GR3276764-5	1:100		Alexa Fluor™ Plus 647 goat anti-rabbit	Thermo Fisher Scientific	A32733	WC310044	1:200	2
6	**CD20**	TRITC	L26	CellMarque	120M-86	109377	1:700	8	Alexa Fluor™ Plus 555 goat anti-mouse	Thermo Fisher Scientific	A32727	WL333735	1:100	2
	**PD-1**	Cy5	EPR4877(2)	abcam	Ab137132	GR3230470-20	1:350		Alexa Fluor™ Plus 647 goat anti-rabbit	Thermo Fisher Scientific	A32733	WC310044	1:200	2
7	**CD38**	TRITC	SP149	CellMarque	118R-16	99807	1:600	8	Alexa Fluor™ Plus 555 goat anti-rabbit	Thermo Fisher Scientific	A32732	WL332194	1:100	2
	**CD163**	Cy5	MRQ-26	CellMarque	163M-15	123086	1:50		Alexa Fluor™ Plus 647 goat anti-mouse	Thermo Fisher Scientific	A32728	WK331591	1:200	2
8	**CD45RA**	TRITC	HI100	BioLegend	304102	B295482	1:600	4	Alexa Fluor™ Plus 555 goat anti-mouse	Thermo Fisher Scientific	A32727	WL333735	1:100	2
	**CD56**	Cy5	MRQ-56	CellMarque	156R-95	132727	1:200		Alexa Fluor™ Plus 647 goat anti-rabbit	Thermo Fisher Scientific	A32733	WC310044	1:200	2
9	**aSMA**	TRITC	1A4	CellMarque	202M-96	130990	1:200	4	Alexa Fluor™ Plus 555 goat anti-mouse	Thermo Fisher Scientific	A32727	WL333735	1:100	2
	**Vimentin**	Cy5	SP20	abcam	ab16700	30021150090	1:300		Alexa Fluor™ Plus 647 goat anti-rabbit	Thermo Fisher Scientific	A32733	WC310044	1:200	2
10	**HLA-DR**	TRITC	TAL-1B5	Santa Cruz	sc-53319	30021500	1:1500	8	Alexa Fluor™ Plus 555 goat anti-mouse	Thermo Fisher Scientific	A32727	WL333735	1:100	2
	**PD-L1**	Cy5	IHC411	GenomeMe	IHC411-100	CC864835	1:200		Alexa Fluor™ Plus 647 goat anti-rabbit	Thermo Fisher Scientific	A32733	WC310044	1:200	2

Supplementary Table 3: Primary and secondary antibodies used for the 20-plex seqIF™ protocol on COMET™ on NSCLC resections.

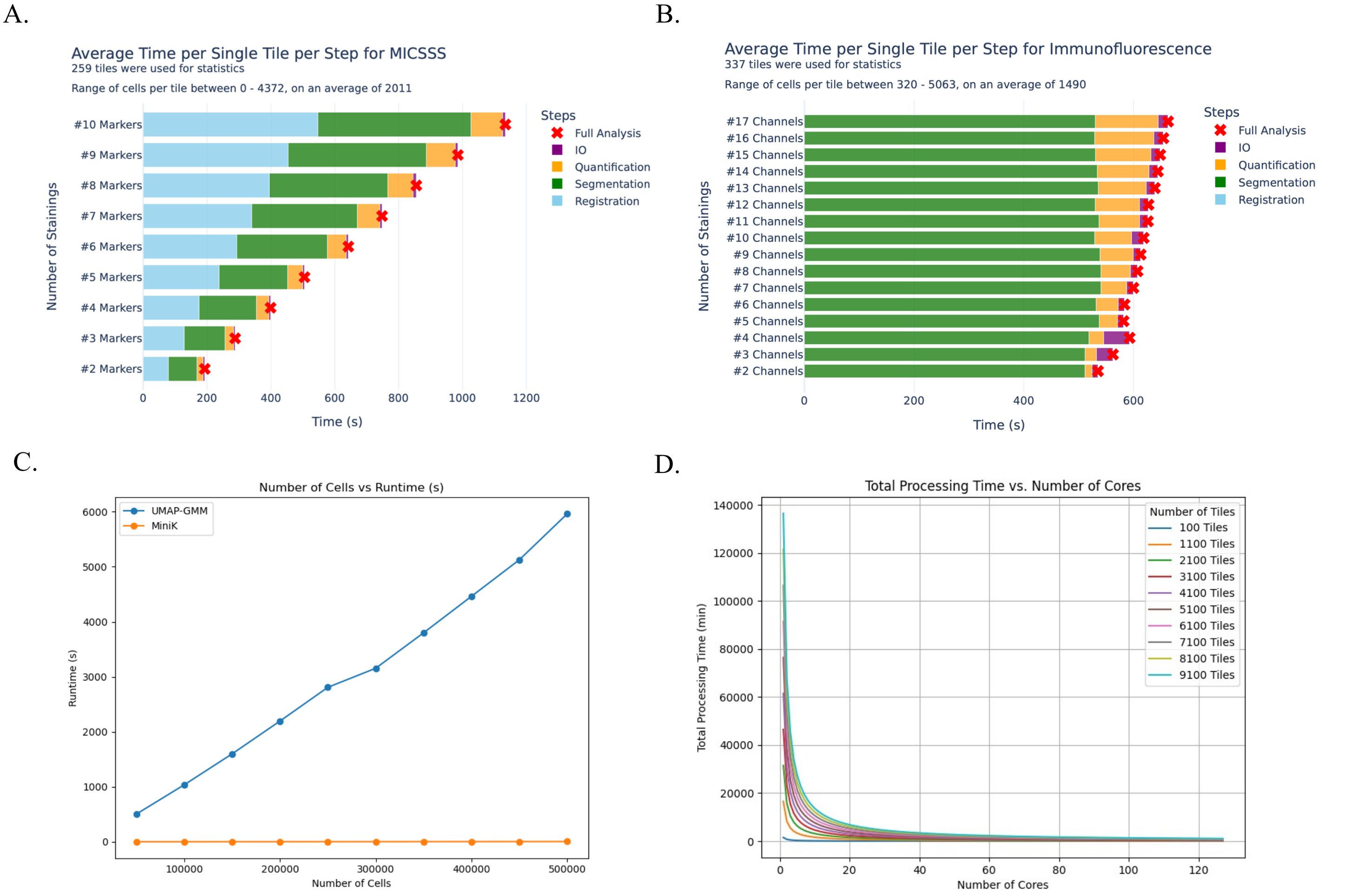

Figure S1: Computational runtimes using MARQO. A) Average runtimes (seconds) per single tile for steps in the MARQO pipeline when analyzing MICSSS, including registration, segmentation, quantification, and clustering (I/O). 259 tiles were used to produce the statistics, with an average cell count of 2011 cells per tile. Each row corresponds to a variable number of markers used in the panel. **B)** Average runtimes (seconds) per single tile for steps in the MARQO pipeline when analyzing immunofluorescence, including segmentation, quantification, and clustering (I/O). 337 tiles were used to produce the statistics, with an average cell count of 1490 cells per tile. Each row corresponds to a variable number of markers used in the panel. **C)** Average runtime (seconds) versus number of total cells analyzed for clustering (I/O) methodologies UMAP-GMM and mini batch K-Means (MiniK). **D)** Total processing times (minutes) versus number of cores for samples with variable number of tiles. All values were generated using one tile per core with 6 GB allotted memory on an Intel Emerald Rapids 8568Y+, 2.3GHz CPU.

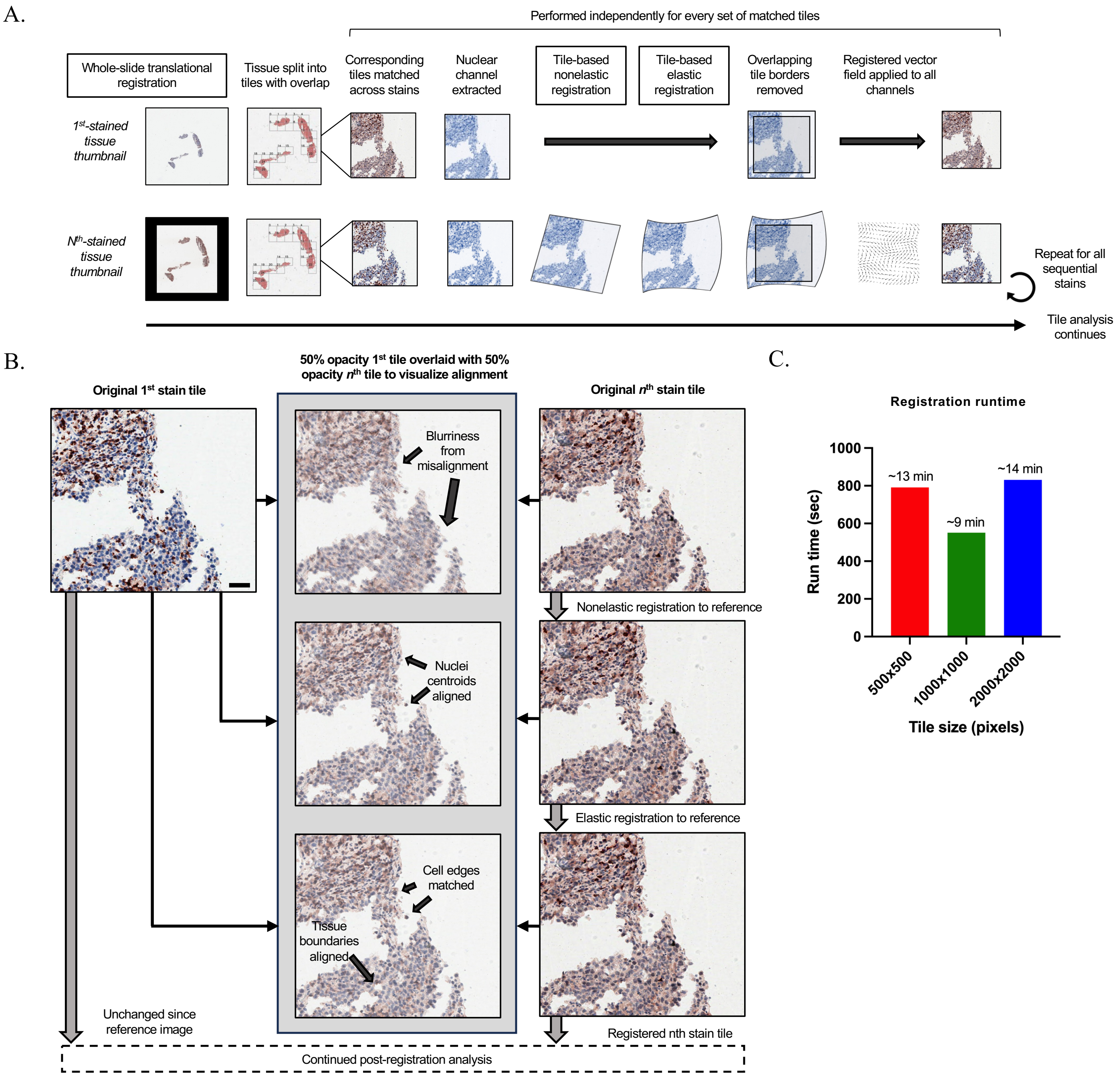

Figure S2: Rigid and elastic registrations methodology with examples. A) MARQO performs an alignment methodology consisting of three distinct registration steps. At first, during the initial user quality control MARQO performs a translational registration of low-resolution thumbnail images, aligning each sequential stain to the first stain thumbnail, which the user can deem as appropriate or revise with tunable, intuitive parameters in the interactive application (see Supplementary Video 1). After tiling of all sequential high-resolution images, MARQO matches corresponding tiles, extracts the nuclear stain, and performs both rigid affine and elastic "B-Spline" registrations on this channel. Tiles were analyzed with overlap which are then removed. The registration vector field from the nuclear channel is applied to the original red-green-blue (RGB) tile so that all tiles are now registered. This process repeats for all tiles and stains, which by default is done in parallel. Analysis per batch of tiles then continues through the MARQO pipeline. **B)** Top row: An example 1st-stained tile (left) and its corresponding nth-stained tile (right) are shown after the initial thumbnail translational registration and tiling steps with an image depicting 50% transparency 1st-stained tile overlaid to 50% transparency nth-stained tile (middle). Note the severe misalignment at the cellular level with dark arrows. Middle row: the nth-stained tile after nonelastic, affine registration (right) and its overlay with the reference tile (middle). Note the aligned cell centroids with some minor misalignment at cell and tissue boundaries with dark arrows. Bottom row: the nth-stained tile after elastic, B-Spline registration (right) and its overlay with the reference tile (middle). Note enhanced cell and tissue boundary alignment with dark arrows. Scale bar = 50μm. **C)** MARQO runtimes (seconds) for various tile sizes (pixels) stained via MICSSS with ten markers, run on a single core with 6 GB allotted memory on an Intel Emerald Rapids 8568Y+, 2.3GHz CPU.

A.

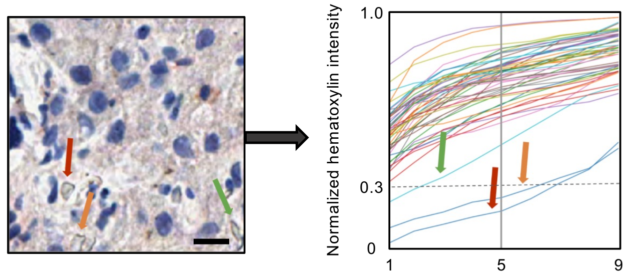

Example image with red blood cells (arrows)

Percentile of pixels within each cell's nuclear boundaries

B.

RBCs manually counted	RBCs eliminated by MARQO (Hit)	RBCs not eliminated by MARQO (Miss)
682	679 (99.6%)	3
3966	3944 (99.5%)	22
1118	853 (76.3%)	265
2820	2117 (75.1%)	703
747	745 (99.7%)	2

C.

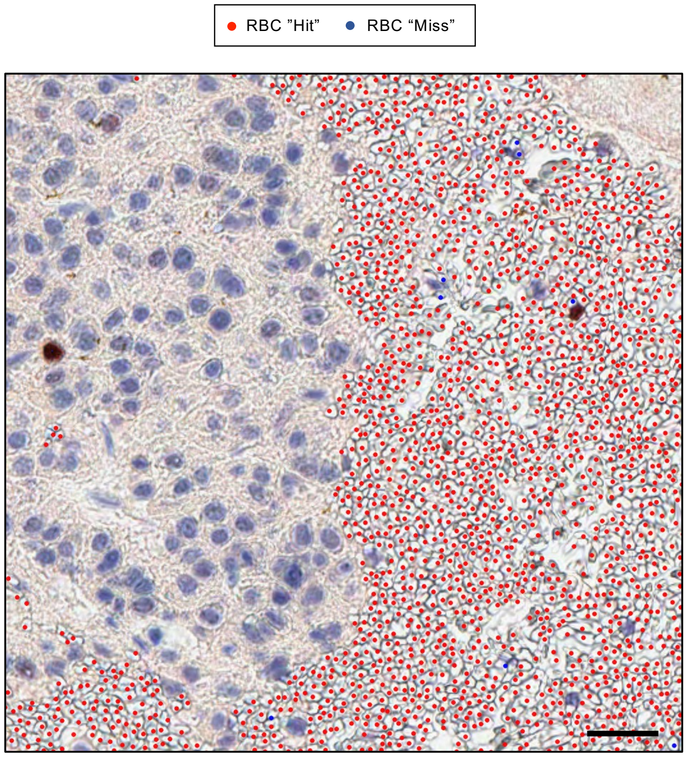

D.

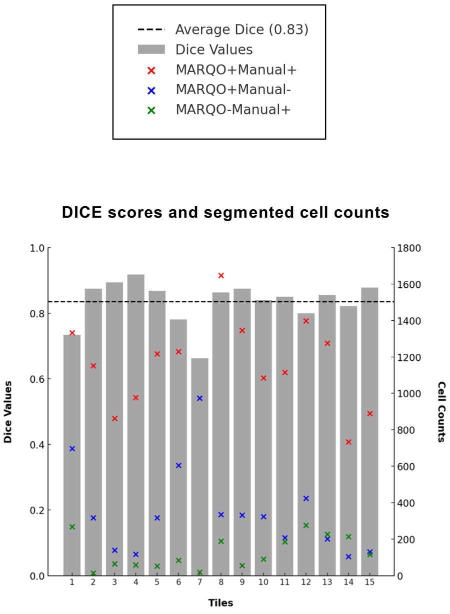

Figure S3: MARQO segmentation performance. A) Example image depicting red blood cells (RBC), shown by arrows. Adjacent graph plots the hematoxylin nuclear counterstain intensity for each cell along all its pixels' percentiles. Scale bar = 10μm. **B)** Contingency table listing total RBCs manually counted by the pathologist (left), total RBCs filtered out and removed by MARQO ("Hit") along with respective percentage of total RBCs counted (middle), and total RBCs not eliminated by MARQO ("Miss") (right). **C)** Example tile (median performance tile shown) that the pathologist used to quantify RBCs, with overlaid centroids plotted. Red points are considered "Hits" and blue points are considered "Misses" by MARQO's RBC filtering algorithm. Scale bar = 30μm. **D)** *Left axis:* Sørensen–Dice coefficient (DICE score) determined per tile used comparing the pathologist's manually selected cells to MARQO's automated composite segmentation using a Point in Polygon (PiP) strategy. A dashed horizontal line is plotted to depict the average DICE score for all 15 tiles. *Right axis:* Raw cell counts considered "MARQO+, Manual+" as red, "MARQO+, Manual-" as blue, and "MARQO-, Manual+" as green plotted versus tile index.

A.

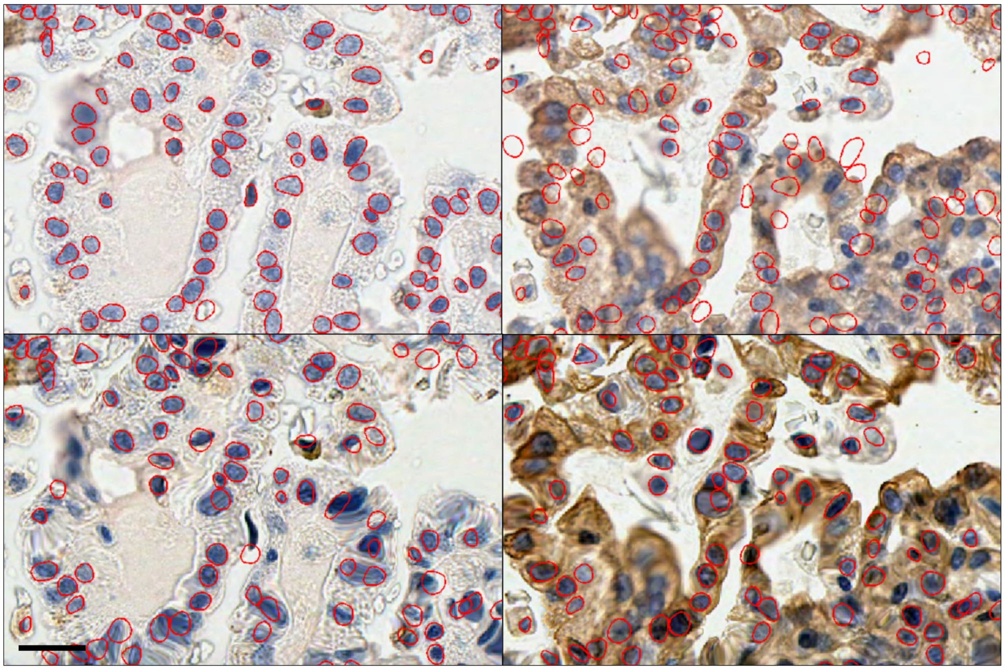

B.

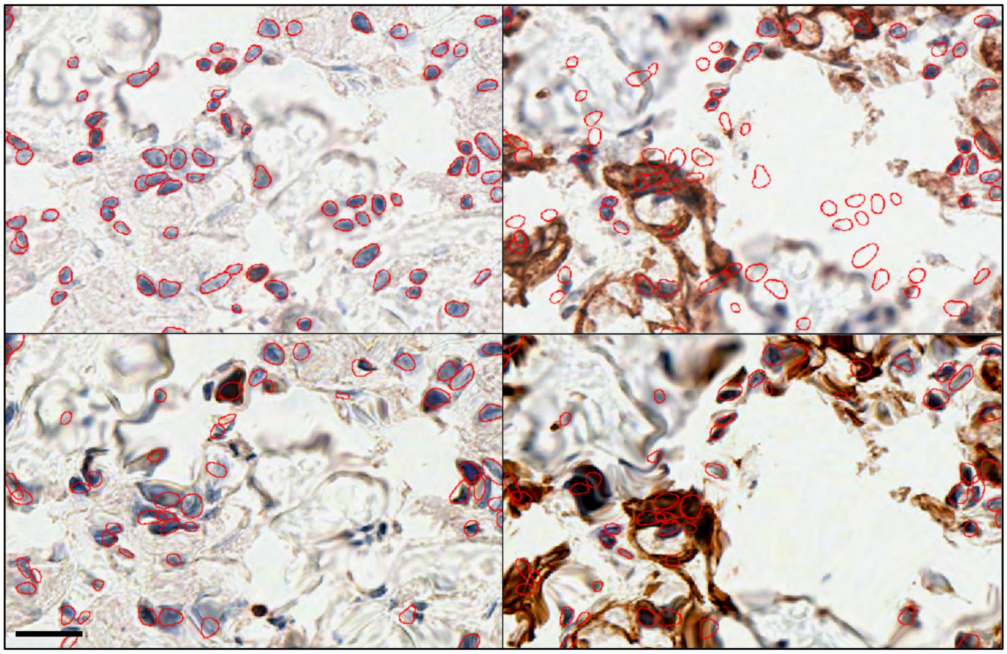

Figure S4: Registration and segmentation performance for QuPath versus MARQO. A) First stain (left) and last stain (right) region of interest for QuPath workflow (top) versus MARQO (bottom) for renal cell carcinoma stained by MICSSS with six total markers. **B)** First stain (left) and last stain (right) region of interest for QuPath workflow (top) versus MARQO (bottom) for pancreatic duct cell carcinoma stained by MICSSS with six total markers. QuPath workflow includes nuclear segmentation on the first stain and applied to sequential stains, with sequential images registered with an affine transformation. The MARQO automated workflow includes composite segmentation and elastic registrations. Scale bars = 20μm.

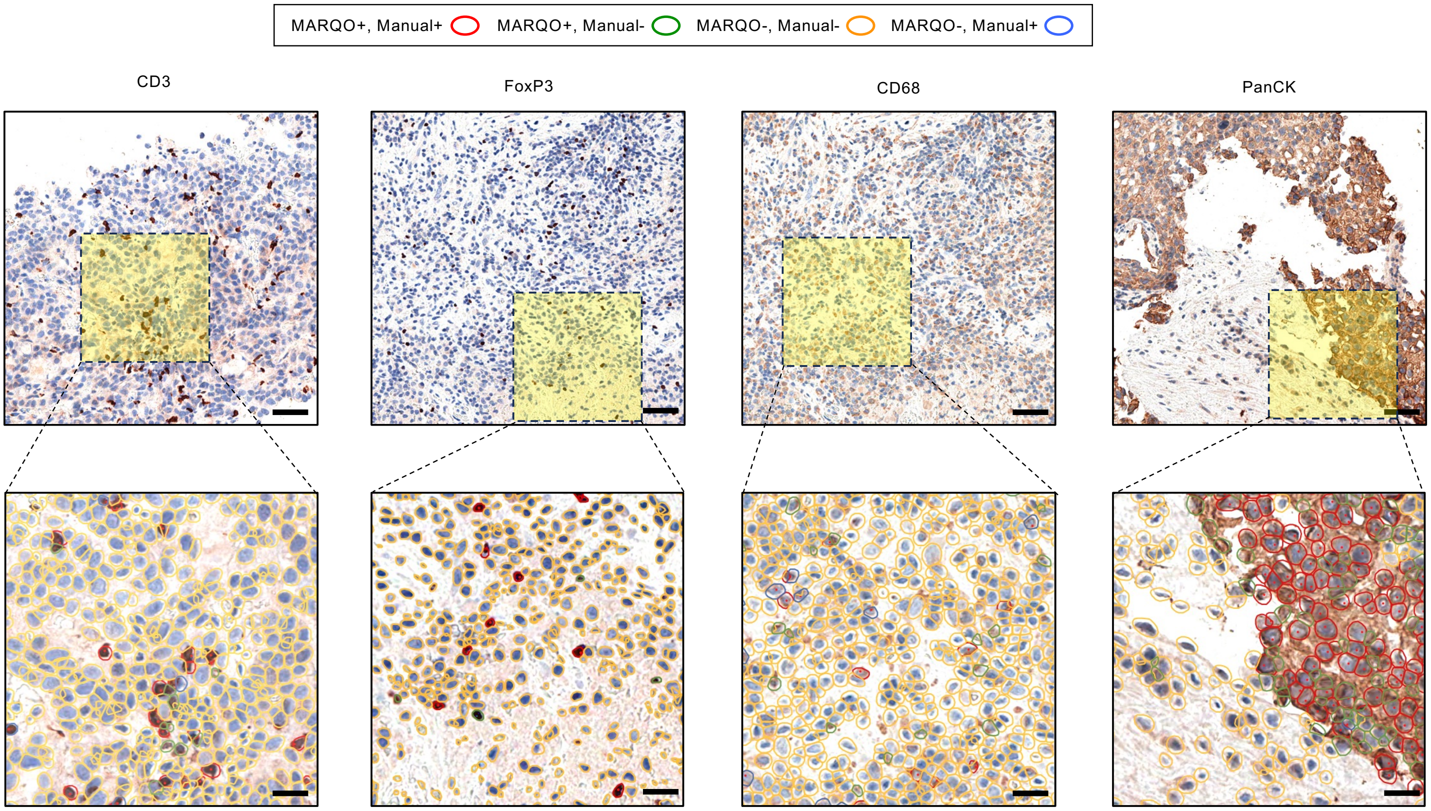

Figure S5: Example tiles used for classification validation. *Top row:* tiles corresponding to the stacked bar graphs provided in Figure 3E across the markers CD3, FoxP3, CD68, and PanCK chosen for classification validation with the pathologist. Scale bars = 55 μm. *Bottom row:* Cropped tiles with areas corresponding to yellow squares overlaid to top row tiles and cell boundaries corresponding to legend. Red signifies cells deemed positive by both MARQO after user quality control and the pathologist. Green signifies cells considered positive by MARQO after user quality control but not the pathologist. Blue signifies cells considered positive by the pathologist but not MARQO after user quality control. Yellow signifies cells considered not positive by both MARQO after user quality control and the pathologist. Scale bars = 20 μm.

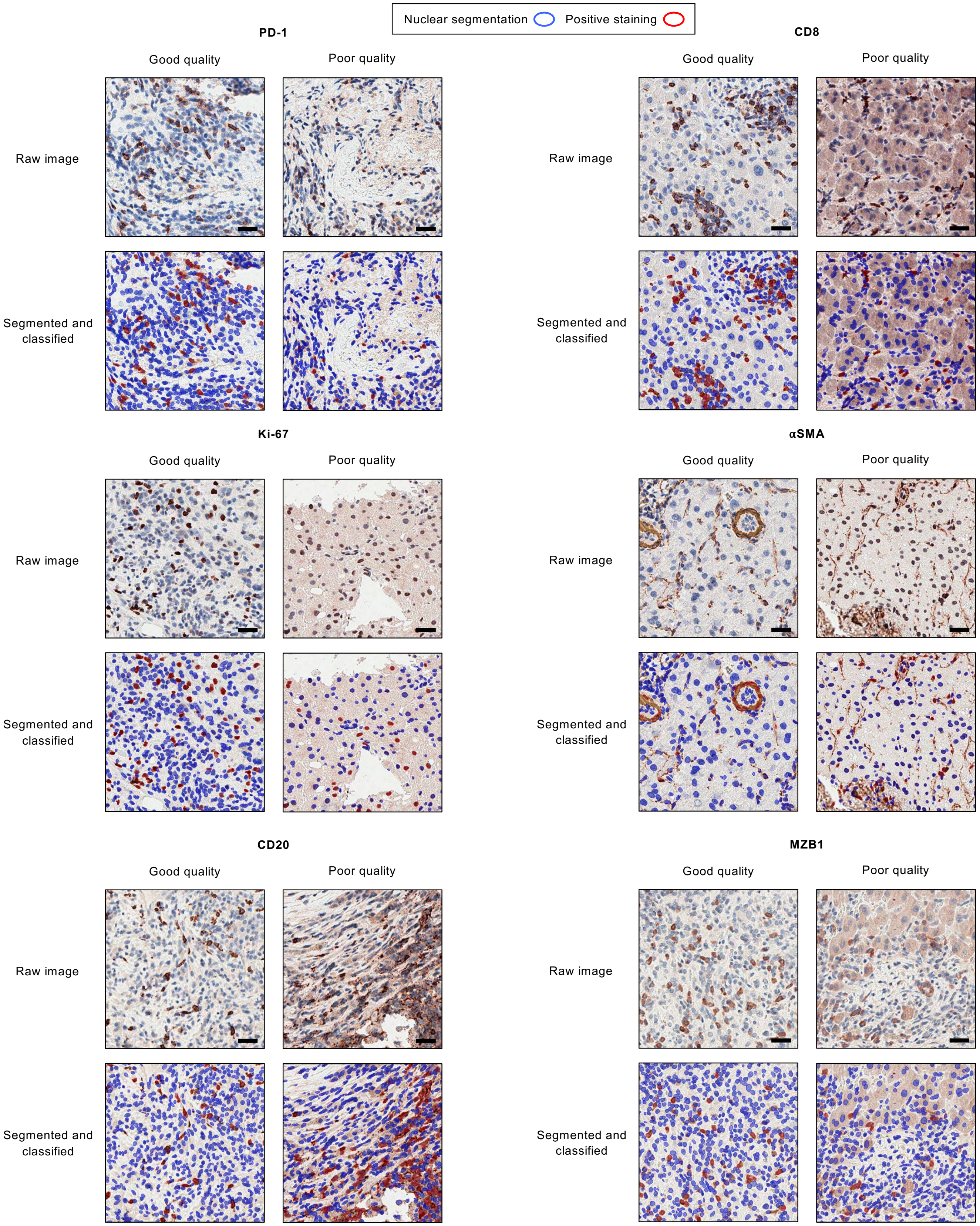

Figure S6: Examples of good- and poor-quality tiles used to test MARQO. These cropped tiles across diverse markers, including PD1, FoxP3, CD3, CD8, Ki-67, αSMA, CD68, NKp46, CD20, and PanCK, were segmented and classified by MARQO and the user with quality control. A pathologist ranked these tiles as “good” (left) and “poor” (right) qualities, defined based on staining, tissue damage, blurriness, or other factors. Scale bars = 30μm.

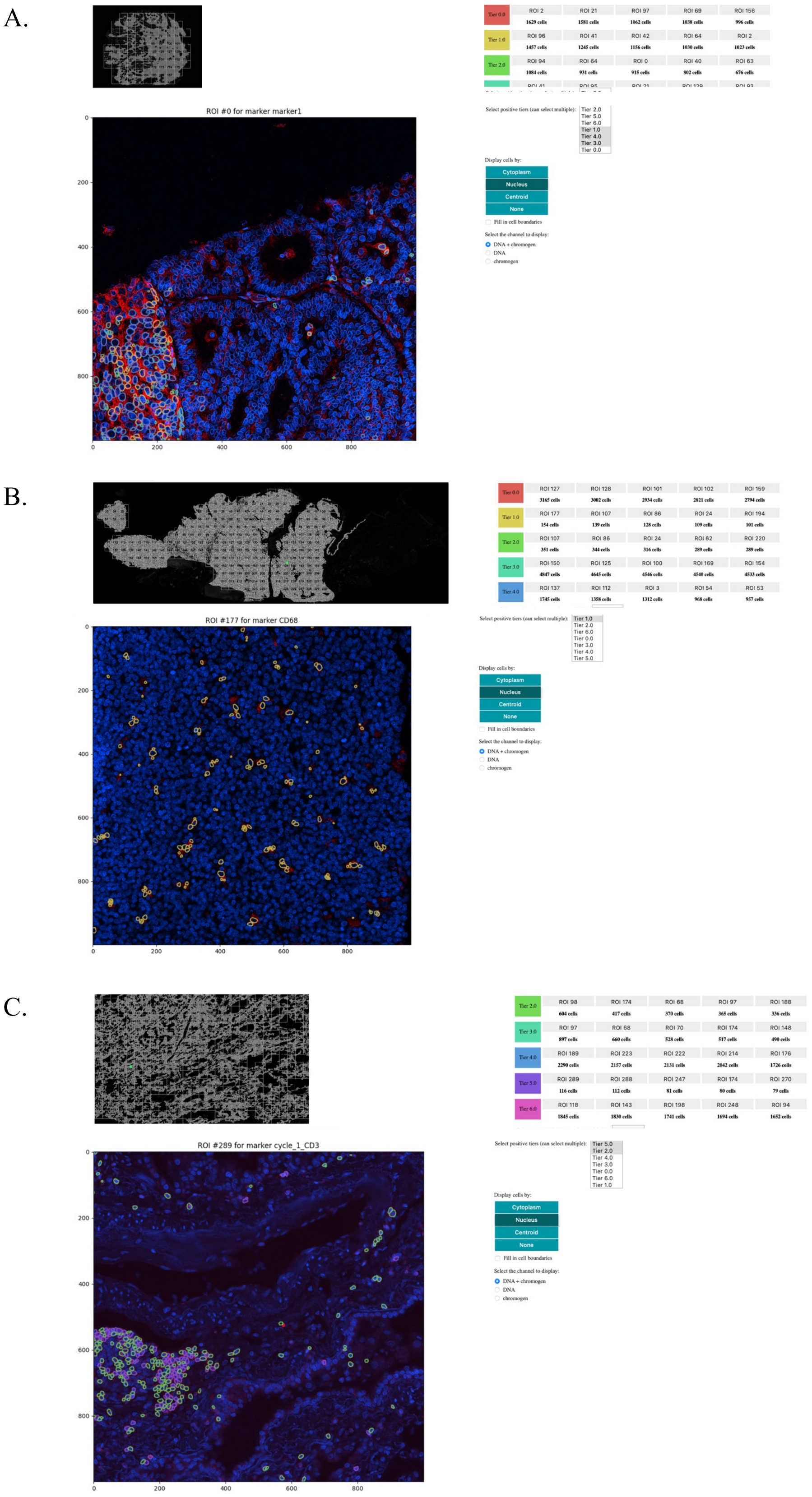

Figure S7: Screenshots of MARQO visualization application for diverse technologies. A) CODEX, **B)** Orion, and **C)** CyCIF example samples visualized in the "Classify cells" module in the MARQO review application.

A.

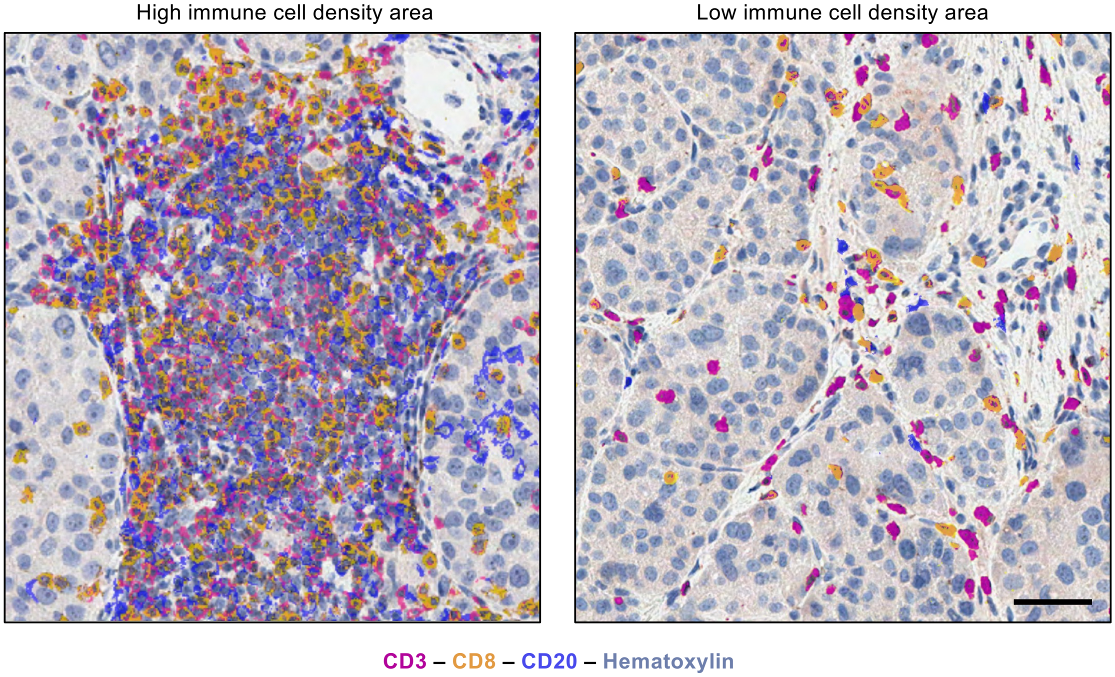

B.

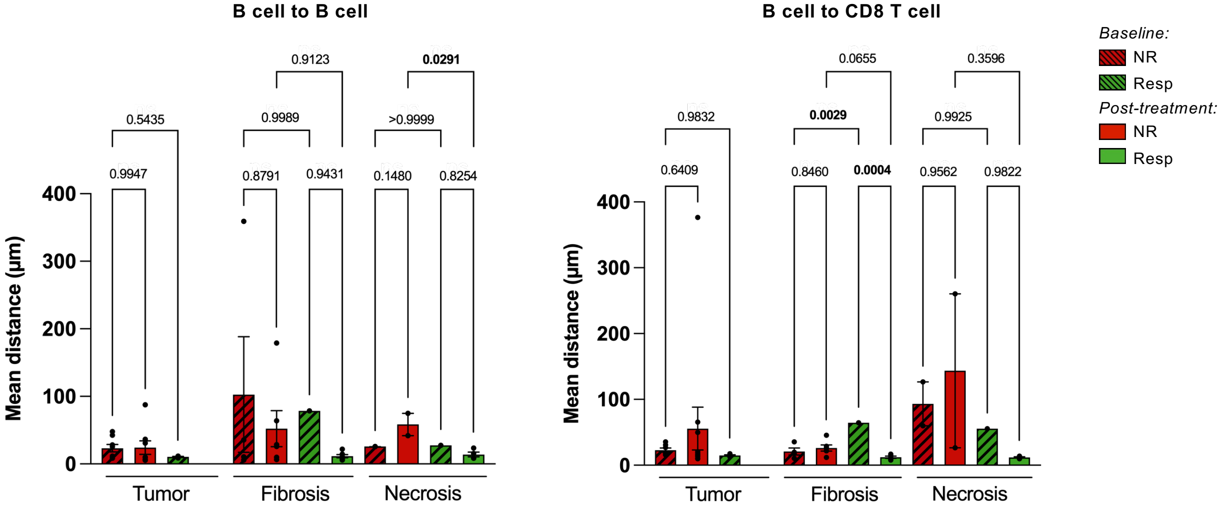

Figure S8: Immune cell proximity visualization and quantification. A) CD3, CD8, and CD20 marker intensity values overlaid to high immune cell density and low immune cell density region of interests for hepatocellular cell carcinoma tissue from responder patients, stained via MICSSS and counterstained with hematoxylin. Scale bars = 50 µm. **B)** Bar plot depicting the means of the shortest distances from B cells to themselves (left) and to CD8 T cells (right) at baseline and post-treatment for NR and Resp within tumor, fibrosis, and necrosis compartments.
