# Supplementary material for: Multiparametric cellular and spatial organization in cancer tissue lesions with a streamlined pipeline
Source: Nat Biomed Eng. 2025 Aug 25;10(3):517–31. doi: 10.1038/s41551-025-01475-9 (PMC13008776; doi:10.1038/s41551-025-01475-9)
Supplement: Supplementary file 2 — Reporting Summary [file 41551_2025_1475_MOESM2_ESM.pdf]

nature portfolio

Corresponding author(s): Dr. Sacha Gnjatic
Last updated by author(s): June 9th, 2025

Reporting Summary

Nature Portfolio wishes to improve the reproducibility of the work that we publish. This form provides structure for consistency and transparency in reporting. For further information on Nature Portfolio policies, see our Editorial Policies and the Editorial Policy Checklist.

Statistics

For all statistical analyses, confirm that the following items are present in the figure legend, table legend, main text, or Methods section.

n/a	Confirmed	
☐	☒	The exact sample size (*n*) for each experimental group/condition, given as a discrete number and unit of measurement
☐	☒	A statement on whether measurements were taken from distinct samples or whether the same sample was measured repeatedly
☐	☒	The statistical test(s) used AND whether they are one- or two-sided<br>*Only common tests should be described solely by name; describe more complex techniques in the Methods section.*
☐	☒	A description of all covariates tested
☐	☒	A description of any assumptions or corrections, such as tests of normality and adjustment for multiple comparisons
☐	☒	A full description of the statistical parameters including central tendency (e.g. means) or other basic estimates (e.g. regression coefficient) AND variation (e.g. standard deviation) or associated estimates of uncertainty (e.g. confidence intervals)
☐	☒	For null hypothesis testing, the test statistic (e.g. *F*, *t*, *r*) with confidence intervals, effect sizes, degrees of freedom and *P* value noted<br>*Give P values as exact values whenever suitable.*
☒	☐	For Bayesian analysis, information on the choice of priors and Markov chain Monte Carlo settings
☐	☒	For hierarchical and complex designs, identification of the appropriate level for tests and full reporting of outcomes
☐	☒	Estimates of effect sizes (e.g. Cohen's *d*, Pearson's *r*), indicating how they were calculated

Our web collection on statistics for biologists contains articles on many of the points above.

Software and code

Policy information about availability of computer code

Data collection	Aperio ImageScope DX visualizer software v.12.3.3 (Leica) was used for MICSSS and singleplex IHC image acquisition, COMET™ Viewer 1.0.2 (Lunaphore) used to visualize COMET OME-TIFF images
Data analysis	QuPath (Open source software) v0.5.1 was used for comparative image analysis.<br>A container with MARQO's GUI to analyze samples locally is available at https://github.com/igorafsouza/MARQO. All source code is available from the corresponding author upon reasonable request. Requests for service to run larger samples on a cluster should be made by contacting the corresponding author. A list of package dependencies present in our environment are listed in our GitHub (marqo-v1.0.0/requirements.txt).

For manuscripts utilizing custom algorithms or software that are central to the research but not yet described in published literature, software must be made available to editors and reviewers. We strongly encourage code deposition in a community repository (e.g. GitHub). See the Nature Portfolio guidelines for submitting code & software for further information.

Data

Policy information about availability of data

All manuscripts must include a data availability statement. This statement should provide the following information, where applicable:

- Accession codes, unique identifiers, or web links for publicly available datasets
- A description of any restrictions on data availability
- For clinical datasets or third party data, please ensure that the statement adheres to our policy

We have provided deidentified sample images per imaging technology on our GitHub repository. Because specimens related to trials require study sponsor permission, the remaining image files we analyzed are available upon request from the corresponding author. Patient co-expression and cell type data are provided in Extended Data Table 1.

Research involving human participants, their data, or biological material

Policy information about studies with human participants or human data. See also policy information about sex, gender (identity/presentation), and sexual orientation and race, ethnicity and racism.

Reporting on sex and gender	Patients were recruited into the clinical trial (ClinicalTrials.gov, NCT03916627, Cohort B) regardless of sex or gender. All patients whom are deemed to be candidates for surgery and fit inclusion/exclusion criteria are offered participation in the clinical trial.<br>All patients at Mount Sinai Hospital undergoing surgery regardless of any prior treatment received are asked to participate in the biorepository, a standard practice at all large academic hospitals.
Reporting on race, ethnicity, or other socially relevant groupings	This study did not use race, ethnicity, or other socially relevant grouping. Patients grouped by their tumor location were obtained in a deidentified fashion without the possibility of being linked with metadata. Patients enrolled in the clinical trial were grouped by treatment response.
Population characteristics	All samples from patients outside the clinical trial were obtained deidentified without the possibility of being linked with metadata. This single-centre, open-label, single-arm, phase 2 trial of cemiplimab monotherapy administered before and after definitive surgery enrolled patients with early-stage hepatocellular carcinoma. Eligible patients were aged 18 years or older and had confirmed resectable hepatocellular carcinoma (Liver Imaging Reporting and Data System [LIRADS] score of 5 on imaging or biopsyproven tumour, or both), an Eastern Cooperative Oncology Group performance status of 0 or 1, and adequate liver function. Patients were enrolled regardless of the underlying cause of hepatocellular carcinoma; patients with a history of hepatitis C virus (HCV) or hepatitis B virus (HBV) infection were eligible for enrolment if viral clearance had occurred or circulating virus was suppressed on HBV-directed therapies. Patients with HIV with an undetectable viral load by PCR and a CD4+ T-cell count higher than 350 cells per µL were also eligible for enrolment.
Recruitment	Criteria of recruitment into the clinical trial (NCT03916627) are listed below, and also found in the protocol which is a supplement to the previously published clinical study (Marron et al, Lancet GI/Hep 2022). Patients enrolled in the biorepository do not have to meet any prespecified inclusion/exclusion criteria, however, retrospectively assessing their charts they all do, also, meet these criteria:<br><br>Key Inclusion Criteria:<br>• Patient must have a known diagnosis of HCC as defined in the protocol<br>• Patient must be willing and able to provide blood samples at the indicated time points<br>• Patient must be willing and able to have excisional or core needle biopsies of tumor prior to initiation of cemiplimab as defined in the protocol<br>• Eastern Cooperative Oncology Group (ECOG) performance status of 0 or 1<br>• Patient is determined to be a surgical candidate for resection of their tumor<br>• Adequate organ and bone marrow function as defined in the protocol<br><br>Key Exclusion Criteria:<br>• Patients who have had any systemic anti-cancer therapy or radiotherapy within 6 months prior to entering the study for their current tumor or a different primary tumor<br>• Patients whose tumor burden, or pace of tumor growth, in the opinion of the Investigator will not permit delaying surgery<br>• Patients who have participated in a study of an investigational agent or an investigational device within 4 weeks of study therapy or 5 half-lives (whichever is longer)<br>• Patients who have had major surgery within 14 days prior to initiation of neoadjuvant therapy<br>• Patients with metastatic disease for whom the intent of surgery would not be curative<br>• Uncontrolled, intercurrent illness as defined in the protocol and as determined by the Investigator<br>• Is receiving systemic steroid therapy or any other form of immunosuppressive therapy within 7 days prior to the first dose of study treatment<br>• Has active autoimmune disease that has required systemic treatment in the past 1 year<br>• Has a known, additional malignancy that is progressing and/or requires active treatment. Exceptions include patients with: basal cell carcinoma of the skin or squamous cell carcinoma of the skin that has undergone potentially curative therapy; in situ cervical or anal cancer; prostate cancer on stable dose of hormonal therapy without rising PSA; breast cancer who have been treated with curative intent, who may be on hormonal therapy.<br>• Encephalitis, meningitis, or uncontrolled seizures in the year prior to informed consent<br>• Uncontrolled infection with human immunodeficiency virus (HIV), HBV or hepatitis C infection (HCV); or diagnosis of immunodeficiency as defined in the protocol

	Tonsil samples were obtained from patients undergoing tonsillectomies, from Leica Biosystems, which purchased FFPE blocks from the Deer Park local hospital. Non-small cell lung cancer (NSCLC) resection samples were obtained from treatment-naive patients undergoing surgical resection at Mount Sinai Hospital (New York, NY). NSCLC, head and neck squamous cell carcinoma (HNSCC), colorectal cancer (CRC), breast cancer (BC), epithelial ovarian cancer (EOC), pancreatic ductal adenocarcinoma (PDAC), glioblastoma (GBM), renal cell carcinoma (RCC), and melanoma (MEL) samples were obtained by the Cooperative Human Tissue Network.
Ethics oversight	Institutional Review Board at the Icahn School of Medicine at Mount Sinai (IRB Human Subjects Electronic Research Applications 18-00407 and 21-01308)

Note that full information on the approval of the study protocol must also be provided in the manuscript.

Field-specific reporting

Please select the one below that is the best fit for your research. If you are not sure, read the appropriate sections before making your selection.

☒ Life sciences ☐ Behavioural & social sciences ☐ Ecological, evolutionary & environmental sciences

For a reference copy of the document with all sections, see nature.com/documents/nr-reporting-summary-flat.pdf

Life sciences study design

All studies must disclose on these points even when the disclosure is negative.

Sample size	With 21 patients in cohort B, the cohort will test a null hypothesis of poor overall response of 5% or less versus an alternative hypothesis of a promising response rate of 20% or more, at a 10% one-sided significance level and 80% power. If the number of pathological responders is 3 or more in a cohort, the null hypothesis is rejected and the treatment for that cohort is recommended for further study. The additional patient samples size that have been analyzed in this manuscript was determined as sufficient for the validation of the different MARQO application.
Data exclusions	Patients were excluded from enrolment if they had metastatic disease, if the surgery was not expected to be curative, or if they had a known additional malignancy requiring active treatment. Patients could not be receiving chronic systemic immunosuppression or have active autoimmune disease requiring systemic treatment in the past year, except for patients with endocrinopathies on hormone replacement therapy. Pregnant women and patients who had undergone a transplant were excluded, as were any patients with a history of CNS or pulmonary inflammatory conditions.
Replication	Reproducibility measures are not applicable in this context, as the study represents a discrete and completed clinical trial. All analyses were conducted on a single, defined cohort of patients with HCC treated with cemiplimab. Additional cohorts incorporating immunodynamic interventions to the cemiplimab treatment are currently being enrolled, but these are part of separate ongoing studies and will be reported in future publications.
Randomization	The clinical trial was a single-arm study with no randomization. Other sample obtainment and assignment was random.
Blinding	Blinding was not performed; the clinical trial was a open-label single-arm study.<br>Regarding the other samples, blinding was not relevant as each sample was assigned based on the imaging technology and direct comparison required for MARQO validation.

Reporting for specific materials, systems and methods

We require information from authors about some types of materials, experimental systems and methods used in many studies. Here, indicate whether each material, system or method listed is relevant to your study. If you are not sure if a list item applies to your research, read the appropriate section before selecting a response.

Materials & experimental systems

n/a	Involved in the study	
☐	☒	Antibodies
☒	☐	Eukaryotic cell lines
☒	☐	Palaeontology and archaeology
☒	☐	Animals and other organisms
☐	☒	Clinical data
☒	☐	Dual use research of concern
☒	☐	Plants

Methods

n/a	Involved in the study	
☒	☐	ChIP-seq
☒	☐	Flow cytometry
☒	☐	MRI-based neuroimaging

Antibodies

Antibodies used

All antibodies are commercially available and have been validated by the vendor for the use of immunohistochemistry or immunofluorescence. Data are available on the manufacturer's website.

Validation

Validation Statements MICSSS:
CD3 LN10 Leica Biosystems: Immunohistochemical analysis of paraffin-embedded human skin with mycosis fungoides (website)
PDL1: Immunohistochemical analysis of paraffin-embedded human non small cell lung carcinoma (website)
PD1: Immunohistochemical analysis of paraffin-embedded human tonsil (website)
CD8: Immunohistochemical analysis of paraffin-embedded human tonsil (website)
CD3 2GV6 Ventana: Immunohistochemical analysis of paraffin-embedded human tonsil (website)
PanCK: Immunohistochemical analysis of paraffin-embedded human tonsil (website)
FoxP3: Immunohistochemical analysis of paraffin-embedded human tonsil (website)
Ki-67: Immunohistochemical analysis of paraffin-embedded human tonsil (website)
aSMA: Immunohistochemical analysis of paraffin-embedded human liver (website)
CD68: Immunohistochemical analysis of paraffin-embedded human tonsil (website)
CD20: Immunohistochemical analysis of paraffin-embedded human tonsil (website)
MZB1: Immunohistochemical analysis of paraffin-embedded human lymph node (website)

Validation Statements Lunaphore:
CD11c: Immunohistochemical analysis of paraffin-embedded human tonsil (website)
LAG-3: Immunohistochemical analysis of paraffin-embedded human tonsil (website)
CK: Immunohistochemical analysis of paraffin-embedded human tonsil (website)
FoxP3: Immunohistochemical analysis of paraffin-embedded human tonsil (website)
CD3: Immunohistochemical analysis of paraffin-embedded human tonsil (website)
CD8: Immunohistochemical analysis of paraffin-embedded human tonsil (website)
CD11b : Immunohistochemical analysis of paraffin-embedded human spleen (website)
Ki-67 : Immunohistochemical analysis of paraffin-embedded human tonsil (website)
CD68 : Immunohistochemical analysis of paraffin-embedded human tonsil (website)
CD4 : Immunohistochemical analysis of paraffin-embedded human tonsil (website)
CD20 : Immunohistochemical analysis of paraffin-embedded human tonsil (website)
PD-1 : Immunohistochemical analysis of paraffin-embedded human tonsil (website)
CD38 : Immunohistochemical analysis of paraffin-embedded human bone marrow (website)
CD163 : Immunohistochemical analysis of paraffin-embedded human placenta (website)
CD45RA : SeqIF™ (sequential immunofluorescence) staining on formalin-fixed paraffin-embedded human pancreatic carcinoma (website)
CD56 : Immunohistochemical analysis of paraffin-embedded human pancreas (website)
aSMA : Immunohistochemical analysis of paraffin-embedded human colon (website)
Vimentin : Immunohistochemical analysis of paraffin-embedded human melanoma (website)
HLA-DR : Immunohistochemical analysis of paraffin-embedded human tonsil (website)
PD-L1 : Immunohistochemical analysis of paraffin-embedded human lung adenocarcinoma (website)
Alexa FluorTM Plus 555 goat anti-rabbit : Immunofluorescence analysis of MCF 10A (positive model) and T-47D (negative model) cells stained with Vimentin Polyclonal Antibody (Product # PA5-27231) (website)
Alexa FluorTM Plus 647 goat anti-mouse : Immunofluorescence analysis of SH-SY5Y (positive model) and T-47D (negative model) cells stained with Nestin Monoclonal Antibody (10C2), eBioscience™ (Product # 14- 9843-80) (website)
Alexa FluorTM Plus 555 goat anti-mouse : Immunofluorescence analysis of SH-SY5Y (positive model) and T-47D (negative model) cells stained with Nestin Monoclonal Antibody (10C2), eBioscience™ (Product # 14- 9843-80) (website)
Alexa FluorTM Plus 647 goat anti-rabbit : Immunofluorescent analysis of ZO-1 in A549 cells (website)

Clinical data

Policy information about clinical studies
All manuscripts should comply with the ICMJE guidelines for publication of clinical research and a completed CONSORT checklist must be included with all submissions.

Clinical trial registration

NCT03916627

Study protocol

https://clinicaltrials.gov/ct2/show/NCT03916627?term=cemiplimab&cond=HCC&cntry=US&state=US%3ANY&city=New+York&draw=2&rank=1

Data collection

Patients were recruited between June 14, 2018 and Nov 25, 2020 at Mount Sinai Hospital, New York. Samples used for this study were also acquired between June 14, 2018 and December 28, 2020. All patients whom are deemed to be candidates for surgery and fit inclusion/exclusion criteria are offered participation in the clinical trial. All patients treated "off-label" prior to the trial opening were enrolled onto the institutional biorepository IRB-approved informed consent.

Outcomes

Primary Outcome Measures:
1. Major pathologic response (MPR) at time of surgery for the NSCLC cohorts
   [Time Frame: At time of surgery]
   Cohorts A1, A2, A3
2. Significant tumor necrosis (STN) at time of surgery is the primary endpoint for the HCC cohorts
   [Time Frame: At time of surgery]

Cohort B, B2
3. Major treatment effect (MTE) at time of surgery is the primary endpoint for the HNSCC cohort
[Time Frame: At time of surgery]
Cohort C

Secondary Outcome Measures:
1. Delay to surgery
[Time Frame: Surgery > 28 days following the end of the cycle of last dose of cemiplimab]
Defined as surgery > 28 days following the end of the cycle of last dose of cemiplimab in the neoadjuvant period.
2. Event-free survival (EFS)
[Time Frame: Up to 60 months following surgery]
Defined as the time from the first dosing of cemiplimab (SBRT for cohort B2) to the date of disease progression that precluded definitive surgery, or recurrence of tumor after successful surgery, or death from any cause.
3. Disease-free survival (DFS)
[Time Frame: Up to 60 months following surgery]
Defined as the time from date of surgery until recurrence of tumor or death from any cause after successful surgery and recovery.
4. Overall response rate (ORR)
[Time Frame: Up to 60 months following surgery]
Defined as the percent of patients with a complete response (CR) or partial response (PR) documented by the Investigator per RECIST 1.1 as described in the protocol.
5. Overall survival (OS)
[Time Frame: Up to 60 months following surgery]
Defined as the time from the first dosing of cemiplimab (chemotherapy for cohort A3 and SBRT for cohort B2) to date of death for any reason.
6. OS rate
[Time Frame: 12 months]
7. OS rate
[Time Frame: 18 months]
8. OS rate
[Time Frame: 24 months]
9. OS rate
[Time Frame: 36 months]
10. OS rate
[Time Frame: 48 months]
11. OS rate
[Time Frame: 60 months]
12. Incidence of treatment-emergent adverse events (TEAEs)
[Time Frame: Up to 60 months following surgery]
Grade 3 or higher per Common Terminology Criteria for Adverse Events (CTCAE V5.0).
13. Incidence of irAEs
[Time Frame: Up to 60 months following surgery]
Grade 3 or higher per CTCAE V5.0.
14. Incidence of SAEs
[Time Frame: Up to 60 months following surgery]
Grade 3 or higher per CTCAE V5.0.
15. Incidence of deaths
[Time Frame: Up to 60 months following surgery]
16. Incidence of laboratory abnormalities
[Time Frame: Up to 60 months following surgery]
Grade 3 or higher per CTCAE V5.0.
17. Change in tumor-infiltrating CD8 T-cell density
[Time Frame: Baseline to time of surgery]
Defined as the change from baseline to the time of surgery.

Plants

Seed stocks

Report on the source of all seed stocks or other plant material used. If applicable, state the seed stock centre and catalogue number. If plant specimens were collected from the field, describe the collection location, date and sampling procedures.

Novel plant genotypes

Describe the methods by which all novel plant genotypes were produced. This includes those generated by transgenic approaches, gene editing, chemical/radiation-based mutagenesis and hybridization. For transgenic lines, describe the transformation method, the number of independent lines analyzed and the generation upon which experiments were performed. For gene-edited lines, describe the editor used, the endogenous sequence targeted for editing, the targeting guide RNA sequence (if applicable) and how the editor was applied.

Authentication

Describe any authentication procedures for each seed stock used or novel genotype generated. Describe any experiments used to assess the effect of a mutation and, where applicable, how potential secondary effects (e.g. second site T-DNA insertions, mosiacism, off-target gene editing) were examined.
